# Stereotactic Body Radiotherapy for Multiple Renal Cell Carcinoma Lesions in a Patient With Polycystic Kidney Disease After Partial Nephrectomy

**DOI:** 10.7759/cureus.83080

**Published:** 2025-04-27

**Authors:** Laura Giannini, Miriam Torrisi, Chiara Lucrezia Deantoni, Roberta Tummineri, Andrei Fodor

**Affiliations:** 1 Radiation Oncology, Istituto Di Ricovero E Cura a Carattere Scientifico Ospedale San Raffaele, Milan, ITA

**Keywords:** clear cell renal carcinoma, cyber knife, renal cell carcinoma (rcc), robotic stereotactic radiotherapy, stereotactic radiotherapy (srt)

## Abstract

Renal cell carcinoma (RCC) is the most common malignant tumor of the kidney. Stereotactic body radiotherapy (SBRT) has recently emerged as a promising non-invasive treatment option for patients with localized RCC. However, data on SBRT in cases involving multiple renal lesions and pre-existing chronic kidney disease remain limited.

We report a case of a 69-year-old male with a history of polycystic kidney disease and prior partial nephrectomy for RCC, who was found to have four new renal lesions during follow-up. At diagnosis of relapse, the patient was treated with SBRT using Cybernife®. All four lesions were treated simultaneously, with a total dose of 30 Gy in five consecutive fractions. No acute toxicity was observed. At the last follow-up, 29 months after SBRT, the MRI confirmed the stable disease observed at the previous controls. Renal function remained stable throughout the follow-up period.

This case supports the use of SBRT as a safe and effective alternative to surgery for patients with multiple RCC lesions and compromised renal function. Even with a reduced dose, SBRT achieved long-term disease control and preservation of renal function suggesting its suitability in selected complex cases.

## Introduction

Renal cell carcinoma (RCC) is the eighth most common cancer diagnosed in Europe and its incidence is rising, particularly among patients older than 70 years [1,2]. RCC is the most common tumor of the kidney accounting for 90% of cases. The standard of care for operable patients with primary RCC is surgical resection [3]. However, not all patients are suitable for surgery due to the typically late onset of the disease, which is often associated with comorbidities and an increased perioperative risk [4]. In these patients, ablative techniques, such as cryoablation and radiofrequency ablation, may be effective in selected cases, with treatment supported by prospective, long-term studies [5]. For a long time, radiotherapy was not considered an effective treatment for presumed radioresistance. However, this paradigm has changed recently with the advent of stereotactic ablative radiotherapy (SBRT), which demonstrated promising local control (LC) while preserving renal function [6,7]. SBRT is a non-invasive, advanced radiotherapy technique, which accurately delivers a very high dose of treatment, using just a few fractions of treatment. This approach has recently demonstrated promising LC, acceptable side effects, and good preservation of renal function in primary RCC, even for tumors up to 10 cm in diameter, including those encroaching the central portion of the kidney [4,7-9]. This report presents a case of a patient presenting multiple RCC lesions who was treated with CyberKnife (Accuray, Sunnyvale, CA, USA), achieving LC, without significant toxicity. Clinical data are drawn from real-world clinical practice and provide insights into the management of RCC several years after the treatment. The purpose of this manuscript is to share our experience in the management of a patient with multiple renal lesions, highlighting the potential role of stereotactic radiotherapy as a non-invasive and effective therapeutic option.

## Case presentation

The patient is a 69-year-old man with a history of RCC. In 2008, he underwent a right partial nephrectomy and adrenalectomy for a pT1 G3 RCC. During follow-up, in 2018, four new lesions were identified in the right kidney at a CT scan. These lesions were initially monitored; however, due to the progressive growth, the patient was referred to our center for further evaluation and treatment. The patient had also polycystic kidney disease, which contributed to a stage IV chronic kidney disease at the time of the first radiotherapy visit. The patient had also a history of Crohn’s disease, high blood pressure, and a splenic artery aneurysm previously treated with stent placement.

The case was first evaluated by a urologist, who recommended total nephrectomy as the treatment of choice. However, this approach carried a very high risk of progression to end-stage renal disease (ESRD), ultimately requiring dialysis.

Subsequently, due to the case's complexity, the patient was discussed into a multidisciplinary tumor board involving radiation oncologists, urologists, medical oncologists, pathologists, and nephrologists. It was agreed that surgery may further worsen renal function, particularly due to the potential for obstructive and infectious complications.

A neoadjuvant medical approach was not recommended based on the limited cytoreductive effect of systemic therapies on primary renal tumors. Additionally, the patient’s pre-existing renal impairment significantly increased the risk of toxicity from anti-angiogenic agents, which could further compromise residual kidney function. Consequently, SBRT to the renal lesions using the CyberKnife system was considered the most appropriate therapeutic approach for this patient.

**Figure 1:**
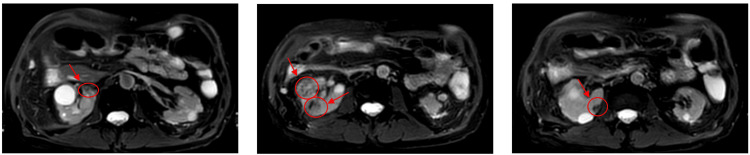
Renal lesions identified on MRI

Prior to treatment, the patient underwent contrast-enhanced abdominal MRI, which revealed four renal lesions in the right kidney: the first located in the inferoposterior third, measuring 29.5 × 27.5 mm; the second in the mid anterolateral third, measuring 24.6 × 23 mm; the third in the upper medial portion of the mid-third, measuring 13 × 11 mm; and the fourth at the medial edge of the upper mid-third, measuring 9 × 9.9 mm (Figure 1). 

Blood tests showed a serum creatinine level of 2.27 mg/dL and an estimated glomerular filtration rate (eGFR) of 29 mL/min. The patient was asymptomatic at the time of evaluation. Following the therapeutic decision at the multidisciplinary tumor board, the patient proceeded with the SBRT workflow. Four fiducial markers were placed using needles inserted into or near the target area by an experienced urologist.

Ten days later, the patient, in the supine position, with arms at his sides, underwent a treatment planning CT simulation scan (with 1.25 mm axial slice thickness) to delineate the four gross tumor volumes (GTVs). A 3 mm isotropic margin was generated to obtain the planning target volume (PTV). No GTV-CTV (clinical target volume) margins were used.

**Figure 2:**
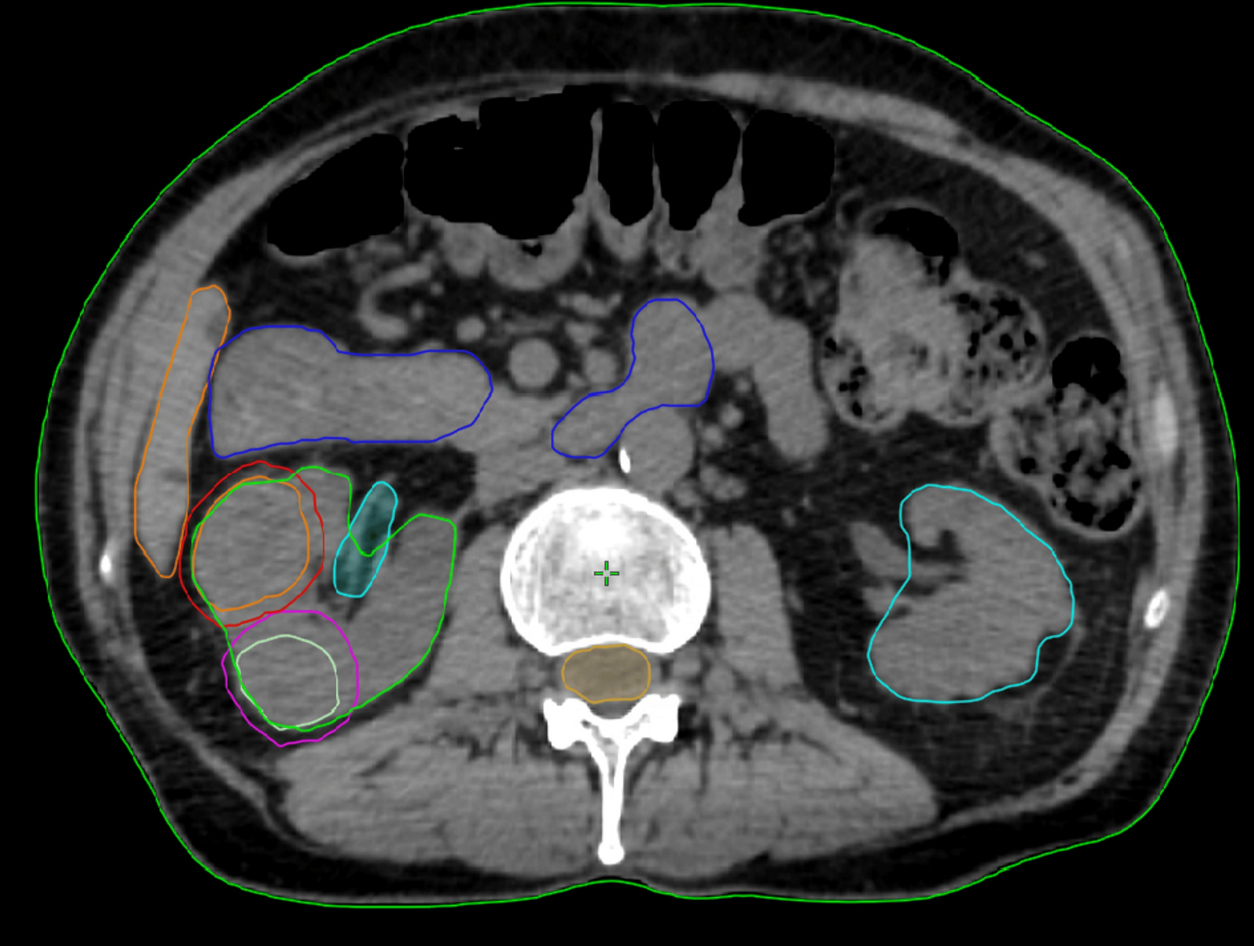
Delineation of GTVs and organs at risk on the planning CT scan GTVs, gross tumor volumes

Organs at risk (spinal cord, ipsilateral and contralateral kidney, small bowel, and liver) were delineated on CT scan and the treatment planning was performed using Accuray's Precision Treatment Planning System, version 3.3.1.2 (Accuray Incorporated, Sunnyvale, CA, USA) (Figure 2) (5). 

The treatment was delivered using a CyberKnifeM6 robotic radiosurgery system with synchrony dynamic respiratory tracking. Fiducial markers placed in the kidney increased the precision of irradiation during treatment.

**Figure 3:**
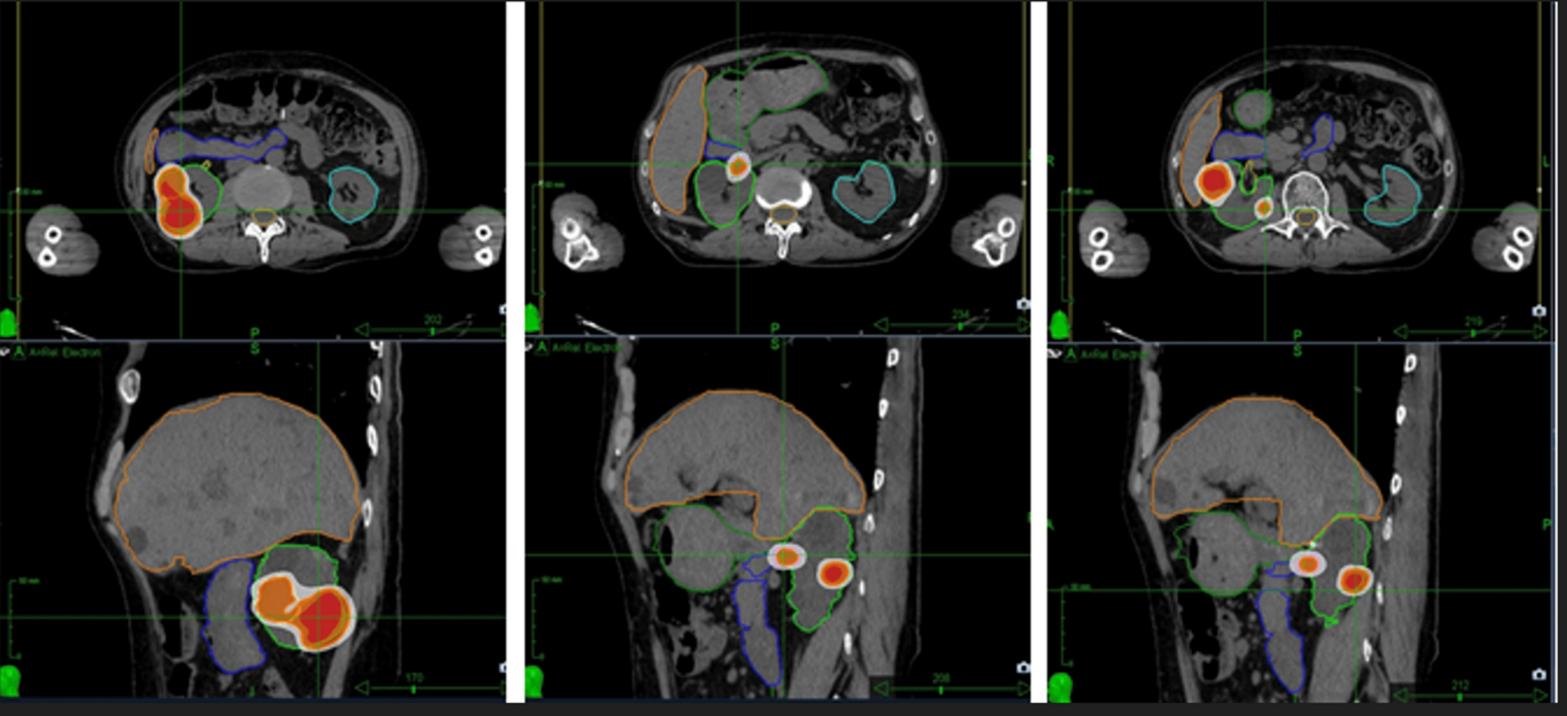
Treatment planning in axial and coronal views for four RCC lesions RCC, renal cell cancer

**Figure 4:**
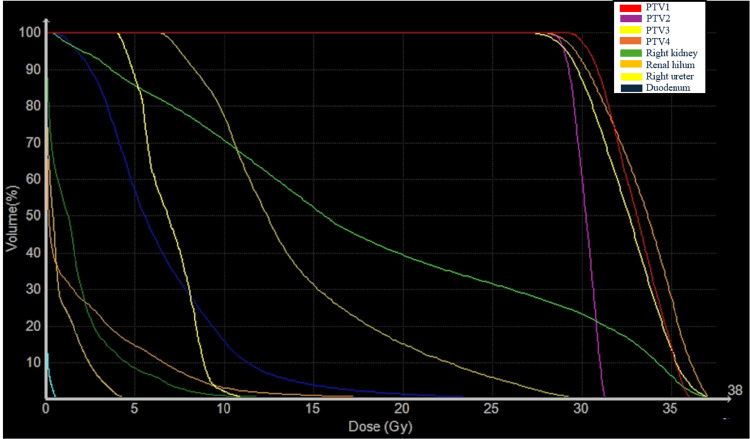
Dose-volume histogram of PTVs and organs at risk PTVs, planning target volumes

The integration of precision robotics and image-guided localization, through the Synchrony respiratory tracking system, compensates for tumor motion, making it a highly effective procedure to reduce the irradiated volume for lesions that move with breathing. An integrated X-ray imaging system acquires orthogonal image pairs before and frequently during treatment to determine the three-dimensional position of the implanted fiducial markers, ensuring real-time motion tracking [10]. The prescription dose was 30 Gy in five fractions, delivered to the 80% isodose on consecutive days (Table 1, Figure 3, Figure 4). 

**Table 1:** Dose and coverage for the four PTVs PTVs, planning target volumes

Name	Dmax (Gy)	Dmean(Gy)	Dmin (Gy)	Coverage (%)	D98 (%)	D95 (%)
PTV1	36.06	33.06	28.43	97.42	99.7	100
PTV2	31.35	30.16	27.34	60.78	86.5	99.2
PTV3	37.5	32.5	24.83	87.25	93.2	97.8
PTV4	37.35	33.38	26.32	92.14	96.3	99.2

Dose constraints were applied according to the recommendations by Rancati et al. and are illustrated in Table 2 [11].

**Table 2:** Dose constraints of organs at risk

Parameter	Organ	Value
Dmax	Small bowel	29.7 Gy
V18 Gy	Small bowel	2.88 cc
V12.5 Gy	Small bowel	10.22 cc
V28 Gy	Small bowel	0.19 cc
Dmax	Stomach	28.23 Gy
V18 Gy	Stomach	0.98 cc
Dmax	Spinal Cord	4.67 Gy

The patient did not receive any adjuvant or symptomatic treatment during SBRT and did not experience acute side effects. Follow-up visits were scheduled every three months with blood tests and contrast-enhanced body MRI. At the last follow-up, 29 months after SBRT, the MRI showed stable disease. The treated lesions exhibit changes in signal intensity on both T1-weighted and T2-weighted sequences, characterized by areas of hypointensity consistent with hemosiderin deposition. Serum creatinine and eGFR remained stable before and after SBRT, with a creatinine level of 1.85 mg/dL and an eGFR of 35 mL/min/1.73 m² at the last follow-up, slightly better than the starting value.

## Discussion

SBRT has proven to be a safe and effective ablative treatment for our patients, ensuring good preservation of renal function and achieving good LC.

Several retrospective and prospective studies have highlighted the clinical value of SBRT in the management of RCC, consistently reporting high rates of LC and low toxicity.

Hanna et al. conducted a prospective phase 2 clinical trial investigating SBRT in patients with biopsy-confirmed radiographically enlarging primary RCC. The study enrolled 16 patients with tumors ≤5 cm in size; 10 patients received 36 Gy in three fractions, while the remaining six received 40 Gy in five fractions. The primary endpoint of the study was LC at one year, assessed using both radiographic criteria and pathologic tumor response. The one-year LC rate was 94%, and all patients demonstrated a significant reduction in tumor growth rates compared to pre-treatment rates. With a median follow-up of 36 months, the LC remained equal to 94%. No grade 2 or grade 3 acute or late toxicities were observed, and none of the patients required dialysis [12].

In the TROG 15.03 FASTRACK II non-randomized phase II trial, 70 patients were enrolled and treated with either 26 Gy in one fraction or 42 Gy in three fractions, depending on tumor size. The study reported 100% LC. Cancer-specific survival at 12 months was 100%, with a favorable toxicity profile and no grade 4 adverse events. Renal function preservation after SBRT was acceptable, with a mean decline in eGFR of 10.8 mL/min/1.73 m² at 12 months and 14.6 mL/min/1.73 m² at 24 months, followed by stabilization thereafter. Only one patient, who had a large central tumor, required dialysis [8].

In 2022, the IROCK reported the five-year outcomes of 190 patients with localized RCC treated with either single- or multi-fraction SBRT. The median dose delivered was 25 Gy in a single fraction and 42 Gy in multiple fractions. The cumulative incidence of local failure was 5.5% at five years and 8.4% at seven years. Patients treated with single-fraction SBRT had significantly lower rates of local failure and higher progression-free survival compared to those who received multi-fraction SBRT. No significant difference was observed in the incidence of grade 2 or worse toxicity between single-fraction SBRT (5%) and multi-fraction SBRT (6%). Compared to baseline, eGFR decreased by a median of 5.5 mL/min/1.73 m² at one year, 10.3 mL/min/1.73 m² at three years, and 14.2 mL/min/1.73 m² at five years. Only seven (4%) patients (two with solitary kidneys) underwent post-SBRT dialysis. One patient developed a grade 4 duodenal ulcer and late grade 4 gastritis [9].

The review of the International Society of Stereotactic Radiosurgery published in 2024 highlights the safety and efficacy of SBRT for RCC. A total of 36 retrospective and prospective trials were analyzed, including 822 patients. The median LC was 94.1% (range 70-100%), five-year progression-free survival was 80.5% (range 72-92%), and five-year overall survival (OS) was 77.2%. SBRT was associated with minimal impact on renal function, with only 3.9% of patients requiring dialysis after treatment. The treatment demonstrated a favorable toxicity profile, with grade ≥3 toxicity observed in only 3.4% of patients [4].

In the SABR meta-analyses by Correa et al., 26 studies were identified (11 prospective trials), including 383 tumors in 372 patients, most of whom were deemed inoperable. Median follow-up, median age, and mean tumor size were 28 months, 70.4 years, and 4.6 cm, respectively. Dose fractionation varied, but 26 Gy in one fraction and 40 Gy in five fractions were the most common. The random-effects estimates were 97.2% for LC, 1.5% for grade 3-4 toxicity, and a post-SABR eGFR decrease of 7.7 mL/min. Six patients (2.9%) with pre-existing renal dysfunction required dialysis. Reported toxicities included mild nausea, fatigue, dermatitis, pyelonephritis, and gastric or duodenal ulcers. No treatment-related mortality was observed [13].

A recent multicenter retrospective study analyzed 144 inoperable patients, treated with SBRT for primary RCC. The prescribed dose was 26 Gy in one fraction or 42 Gy in three fractions. LC probability was 98% at one year and 96% at five years. Median OS was 58 months, and ≥ grade 3 toxicity occurred in 2% of patients, with two requiring dialysis. The median eGFR loss was only 7 mL/min (IQR -17 to 0) [7].

Currently, there is no universally accepted standard for SBRT dose and fractionation in RCC. However, the ISRS consensus recommends delivering an EQD2 of at least 72 Gy, suggesting a potential dose-response relationship. Renal function has been shown to decline linearly with the dose received by the healthy ipsilateral kidney, up to a BED₃ of 100 Gy [4,6].

The decision to prescribe a lower dose (EQD2 of 54 Gy) for our patient was made in order to prioritize renal function preservation and minimize the risk of toxicity, given the presence of multiple lesions and the patient's underlying stage IV chronic kidney disease. Despite the lower dose, good LC was achieved, and renal function remained stable over long-term follow-up. Huang et al. conducted the first meta-analysis specifically investigating the dose-response relationship of SBRT in RCC, including data from 724 patients across 22 studies. Despite a wide range of delivered doses (mean BED₁₀ ranging from 57.1 to 149.5 Gy), no definitive correlation was found between increasing BED and oncologic outcomes such as LC, progression-free survival, or OS. LC rates remained consistently high across all dose levels, with LC estimated at 99% at one year, 98% at two years, and 94% at five years. Notably, no statistically significant association was observed between LC and increasing BED₁₀ at any time point. These findings suggest that the excellent LC achieved across all dose ranges may reduce the necessity for aggressive dose escalation. In line with this, the authors recommend prioritizing the protection of organs at risk over maximizing the target dose, especially in anatomically complex cases or those with adjacent critical structures. Even with partial underdosage or lower prescription doses, SBRT may still provide optimal LC. No association between BED and progression-free survival was observed, which the authors attribute to the predominantly metastatic pattern of progression in RCC. This supports the growing interest in combining SBRT with systemic therapies to more effectively address distant diseases [14].

## Conclusions

In conclusion, SBRT has proven to be a valuable treatment option for our patient with multiple renal lesions from RCC, with contraindications to surgery and pre-existing renal dysfunction. This case supports the growing evidence for the use of SBRT in the management of RCC, demonstrating its effectiveness in achieving LC while preserving renal function. Despite the use of a lower dose (EQD2 of 54 Gy) to minimize toxicity, the patient achieved long-term stable disease, indicating that dose escalation may not be necessary in all cases. Further studies and larger cohorts are needed to fully establish the optimal dose-fractionation schedules for SBRT in complex cases with multiple lesions from RCC.
